# The Effect of the Levonorgestrel-Releasing Intrauterine System on Myometrial Blood Flow in Patients With Heavy Menstrual Bleeding

**DOI:** 10.7759/cureus.66712

**Published:** 2024-08-12

**Authors:** Avantika Gupta, Neha Gangane, Minal Dhanvij, Bishnupriya Moharana, Medha Davile, Shuchita Mundle

**Affiliations:** 1 Obstetrics and Gynaecology, All India Institute of Medical Sciences, Nagpur, Nagpur, IND

**Keywords:** myometrial blood flow, ri and pi of uterine artery, uterine artery doppler, lng-ius, heavy menstrual bleeding, aub

## Abstract

Background

Abnormal uterine bleeding constitutes a vexing issue among female patients, substantially impacting their quality of life. Surgical interventions, particularly hysterectomy, contribute to the psychological, physical, and financial burden on families and, by extension, the healthcare system. Levonorgestrel-releasing intrauterine system (LNG-IUS) represents a conservative management approach and emerges as a beneficial option for affected patients. The present study aims to elucidate color Doppler changes in the uterine artery pre- and post-LNG-IUS insertion.

Objective

The primary objectives encompass an investigation into the variations in Doppler indices (resistance index (RI) and pulsatility index (PI)) within the arcuate and radial branches of the uterine artery, as well as the assessment of endometrial thickness before LNG-IUD insertion, at three months, and six months post-insertion. Secondary outcomes include evaluating changes in pictorial blood assessment chart (PBAC) scores before insertion, at three months, and at six months after LNG-IUS insertion.

Methods

A cross-sectional study was conducted at the Department of Obstetrics and Gynecology at All India Institute Of Medical Sciences (AIIMS), Nagpur. A cohort of 25 women underwent LNG-IUS insertion. The endometrial cavity, RI, and PI of both arcuate and radial arteries were assessed before LNG-IUS insertion and at three and six months after insertion.

Results

The PI of the arcuate artery exhibited minimal alteration over the six-month duration, with a p-value of 0.43. Conversely, the RI demonstrated a statistically significant increase over the same period (p = 0.03). Conversely, the radial artery exhibited no statistically significant changes in either PI or RI (p = 0.39 or 0.13, respectively).

Conclusion

Following six months of LNG-IUS utilization, a substantial reduction in endometrial thickness and menstrual flow was observed, concomitant with an improvement in hemoglobin levels. Notably, the PI of both the arcuate and radial arteries demonstrated no significant change. Although the RI of the arcuate artery increased, its clinical relevance may be limited. Consequently, the observed reduction in menstrual bleeding cannot conclusively be ascribed to diminished blood flow in uterine arteries.

## Introduction

Heavy menstrual bleeding (HMB), as defined, denotes an excessive menstrual blood loss impinging upon a woman's physical, social, emotional, and quality of life [1].

In the research context, it manifests as a score surpassing 100 on a validated pictorial blood assessment chart (PBAC) [2]. The foremost therapeutic recommendation for HMB patients is the levonorgestrel intrauterine system (LNG-IUS) [1]. It is well-established that the localized progesterone impact of LNG-IUS surpasses the systemic effect of orally administered levonorgestrel. However, the precise mechanism underlying this localized effect remains incompletely elucidated. Various studies have yielded disparate findings, with some indicating a reduction in sub-endometrial blood flow and others reporting no alteration in blood flow [2-5]. Notably, Zalel et al. [3] observed a significant decrease in sub-endometrial flow in spiral arteries among 75% of LNG-IUS users, suggesting distinct local progestational effects [2]. Jarvela et al. asserted that LNG-IUS impedes blood flow during the mid-luteal phase [4]. In agreement, Haberal et al. concluded that the uterine artery resistance index increases with LNG-IUD usage [5].

Our hypothesis posits that LNG-IUS induces a reduction in local blood flow, which is evident in increased impedance in the arcuate and radial branches of the uterine artery. This proposed mechanism may elucidate how LNG-IUS mitigates blood loss and potentially account for non-responsiveness to its effects.

Primary study objectives encompass an examination of Doppler indices (resistance index (RI) and pulsatility index (PI)) within arcuate and radial branches of the uterine artery, along with endometrial thickness, both pre-LNG-IUD insertion and at three and six months post-insertion. Secondary outcomes include alterations in PBAC scores before insertion, at three months, and six months post-insertion.

## Materials and methods

Study design: Cross-sectional study

Inclusion criteria: Parous women of age between 20 and 45 years of age and presenting with a complaint of HMB, defined as a score of more than 100 on PBAC, were screened for eligibility for LNG-IUS insertion after a detailed menstrual history, medical history, and gynecological examination. The mandatory tests for workup of HMB (i.e., hemoglobin levels, serum TSH, Pap smear, and pelvic ultrasound) were done for all the patients. A pelvic ultrasound was done in the outpatient department during the same visit to rule out any structural lesions. Endometrial aspiration biopsy using Pipelle’s instrument was done in OPD in women > 40 years or if any high-risk factors for endometrial carcinoma are present such as obesity with diabetes or hypertension, polycystic ovary syndrome (PCOS), endometrial thickness > 12 mm, or family history of endometrial/breast carcinoma.

Exclusion criteria: Patients with genital infection, hormonal pills in the preceding three months, coagulopathy, uterine myoma, vascular malformation of the uterine artery, and genital malignancy were excluded.

Ethical considerations: This study was done as per the ethical standards set by the Institute Scientific Advisory and Ethical Committee for Human Studies, following the 1964 Declaration of Helsinki and its later versions.

Methodology

**Figure 1:**
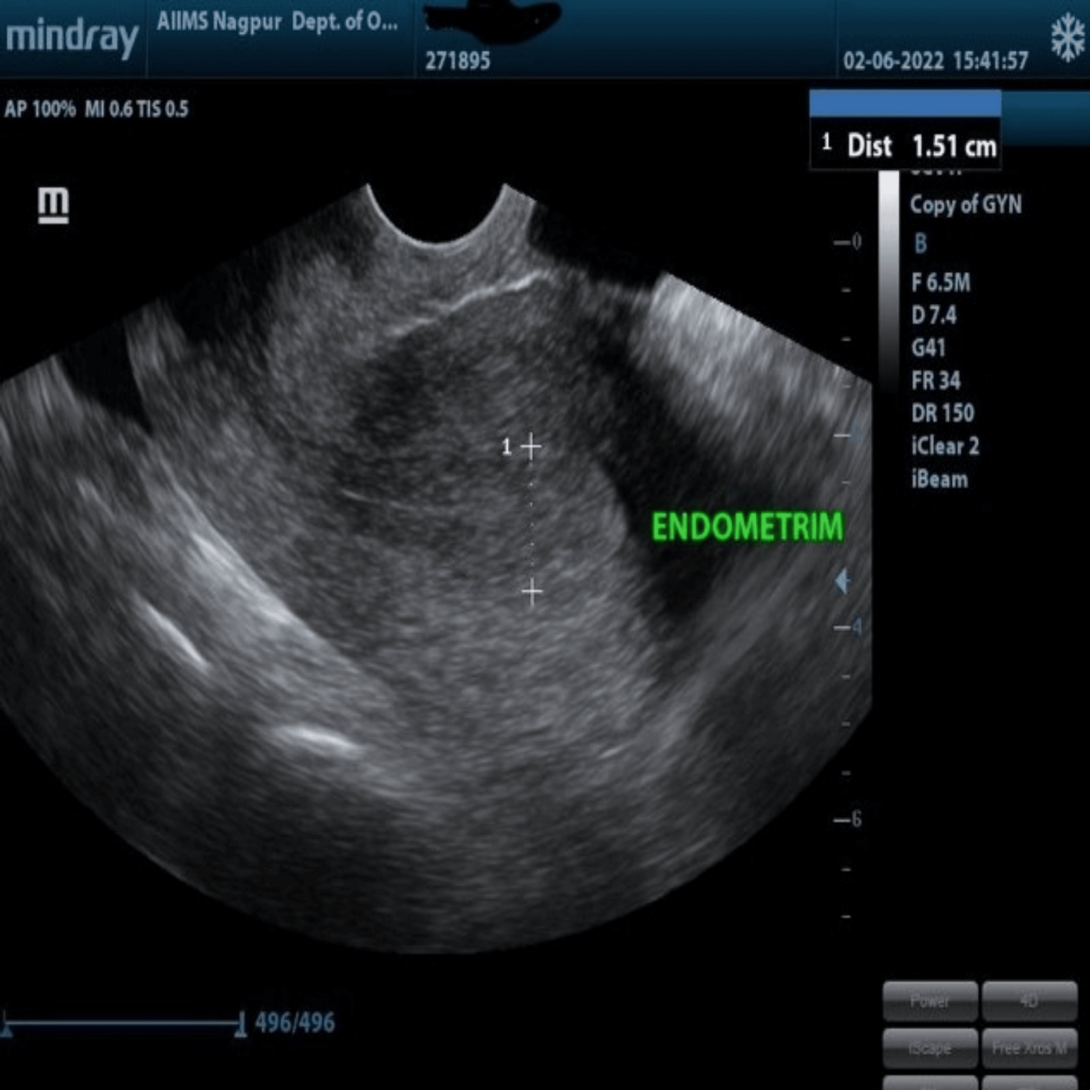
Measurement of the baseline endometrial lining on a 2D gray scale

**Figure 2:**
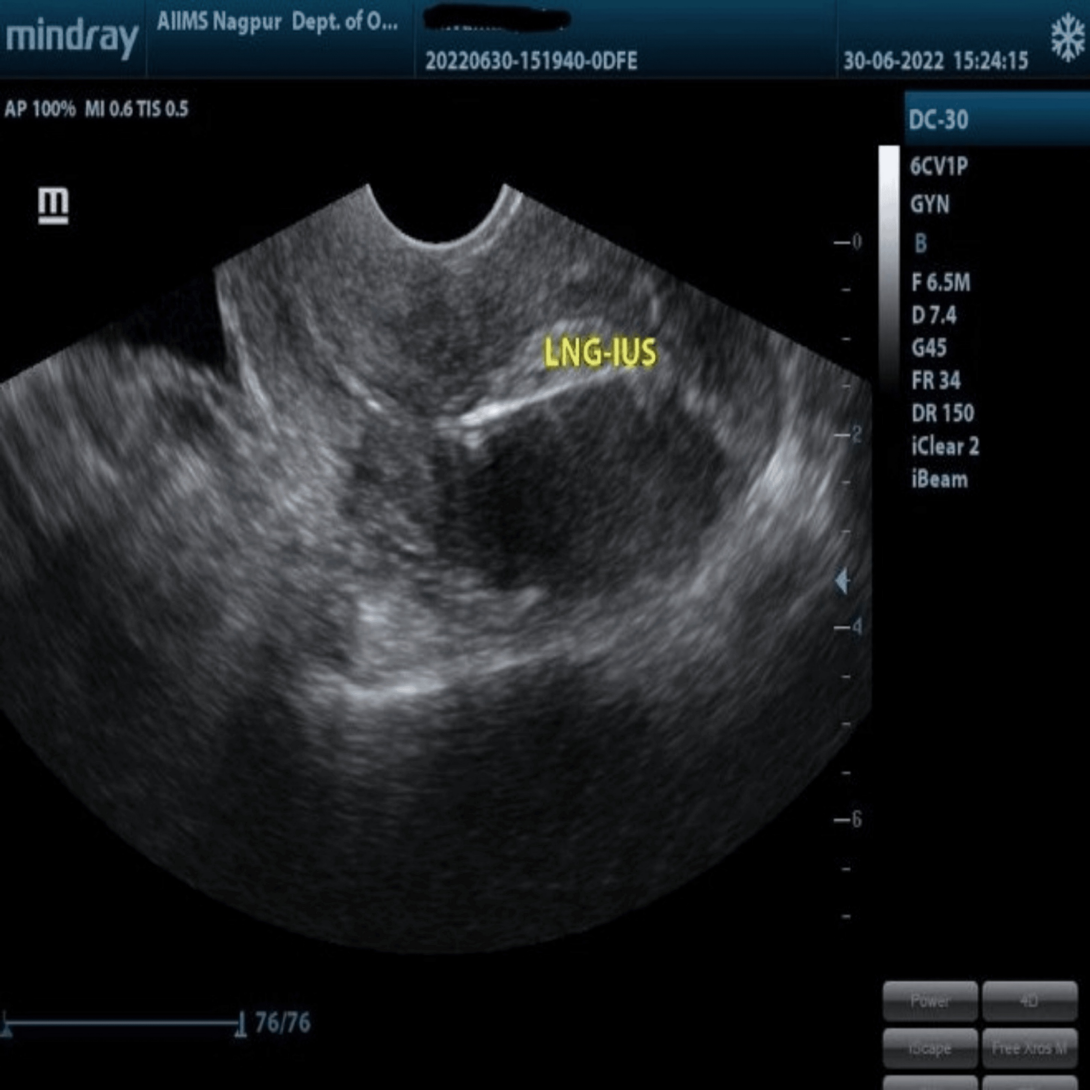
The thickness of LNG-IUS is deducted from the endometrial thickness measurement

A written informed consent was obtained from the patients before enrollment into the study. The scheduled visit was defined as a follow-up at the third and sixth months of LNG-IUS insertion with a margin of seven days to reduce the attrition rate. The patients were asked to come within 3-10 days of menses for LNG-IUS insertion and instructed to avoid intake of nonsteroidal anti-inflammatory drugs (NSAIDs) for 24 hours. Baseline endometrial thickness and uterine artery Doppler indices (PI and RI) were measured just before LNG-IUS insertion using a 6.5 MHz transvaginal probe of an ultrasound machine (Mindray DC-30, India). With the patient in the dorsal position, the transvaginal probe covered with a condom was advanced to the posterior fornix to get a sagittal section of the uterus. Endometrial thickness was measured in 2D B mode as a sub-endometrial region 1 mm parallel to the myometrial-endometrial, at the thickest part in the sagittal section, including both endometrial layers (Figure 1) and LNG-IUS in Figure 2.

**Figure 3:**
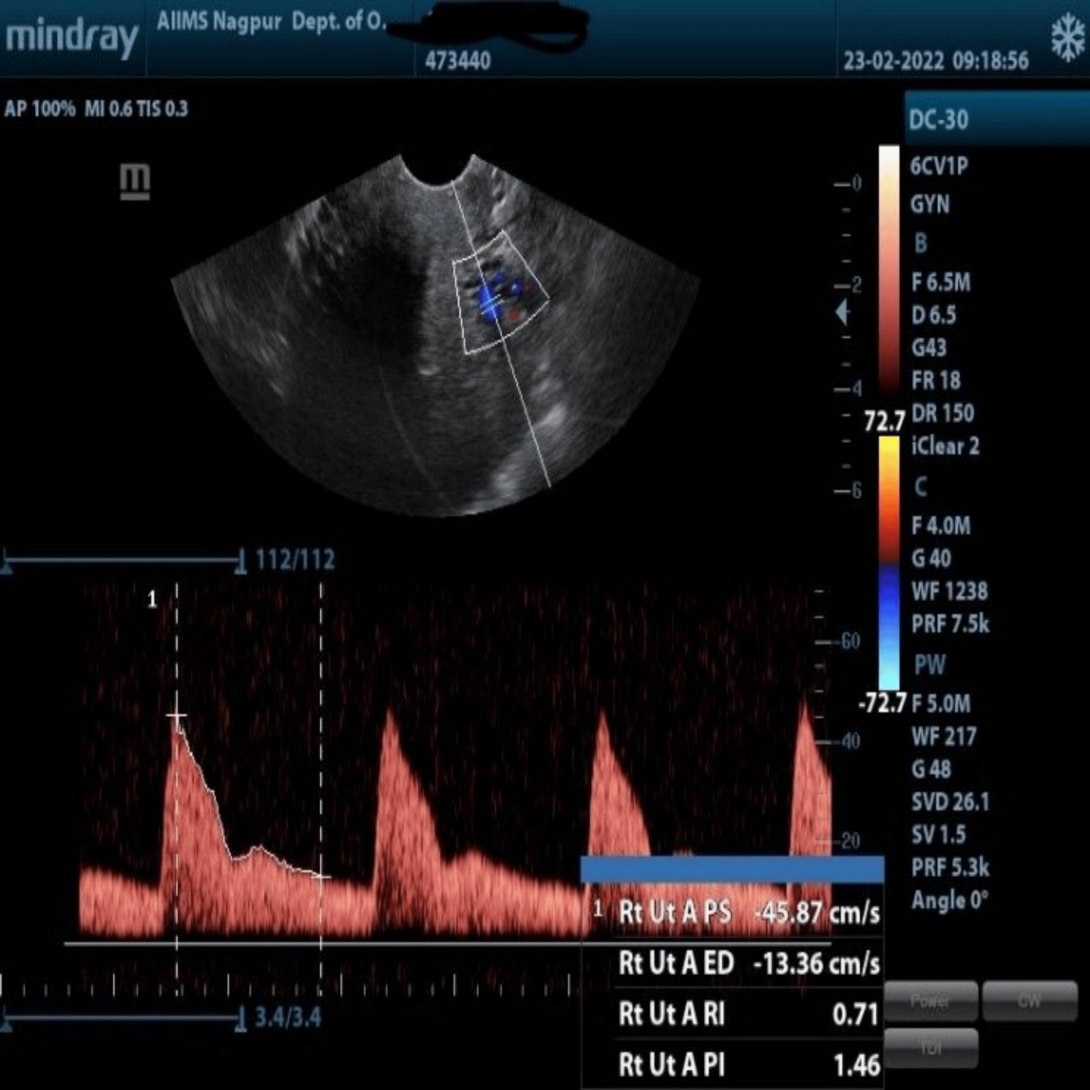
Measurement of the arcuate artery waveform at the outer one-third of the myometrium

Color Doppler was applied to identify the arcuate artery in the outer one-third of the myometrium and radial arteries traversing toward the endometrium. Then, a pulsed wave doppler was applied at a sampling rate of 2 mm with the angle of insonation less than 30 degrees. The settings of the power Doppler sonogram were kept as a high pass filter of 50 Hz, pulsed repetition frequency @ 750 Hz, and moderate to long persistence (Figure 3). The mean PI and RI were determined automatically after obtaining at least three equally sized waveforms. PI is calculated automatically using the formula A-B/mean and RI is calculated as A-B/A (A = peak systolic Doppler shift frequency, B = end diastolic shift frequency, mean = mean maximum Doppler shift frequency). The Doppler study of the uterine artery was performed by a single operator trained in Doppler ultrasound blinded to the patient’s clinical characteristics. All Doppler studies were done for endometrial thickness within 9-11 mm in the outpatient department to avoid diurnal variation in the results.

Outcome Measures

a) The primary outcome of the study was measured as uterine artery Doppler indices (RI and PI) and endometrial thickness before LNG-IUS insertion and at three months and six months after insertion. b) The secondary outcome will be a mean score of PBAC before insertion and at three months and six months after insertion.

Sample Size

To detect a difference of 0.42 in PI with SD of 0.58 of uterine artery before and after insertion, with a 5% level of significance and 90% power, 22 women were needed. Keeping a 10% rate of attrition, 25 women were included in the study. With LNG-IUS, sanitary pads, and Doppler study being made available free of cost to patients and keeping a margin of seven days for follow-up visits, we expect an attrition rate of less than 10% only.

Statistical analysis

Continuous variables (age, BMI, uterine artery PI, and RI, blood loss estimation by PBAC, hemoglobin levels, and endometrial thickness) were expressed as mean with standard deviations or median with inter-quartile range if the data are normally distributed or non-normally distributed, respectively. The mean difference of continuous variables before and after insertion was analyzed using paired t-test/Wilcoxon signed rank sum test or median difference was analyzed using the Friedman test. Categorical variables were expressed as numbers and percentages. P-value < 0.05 was considered to be significant.

## Results

**Figure 4:**
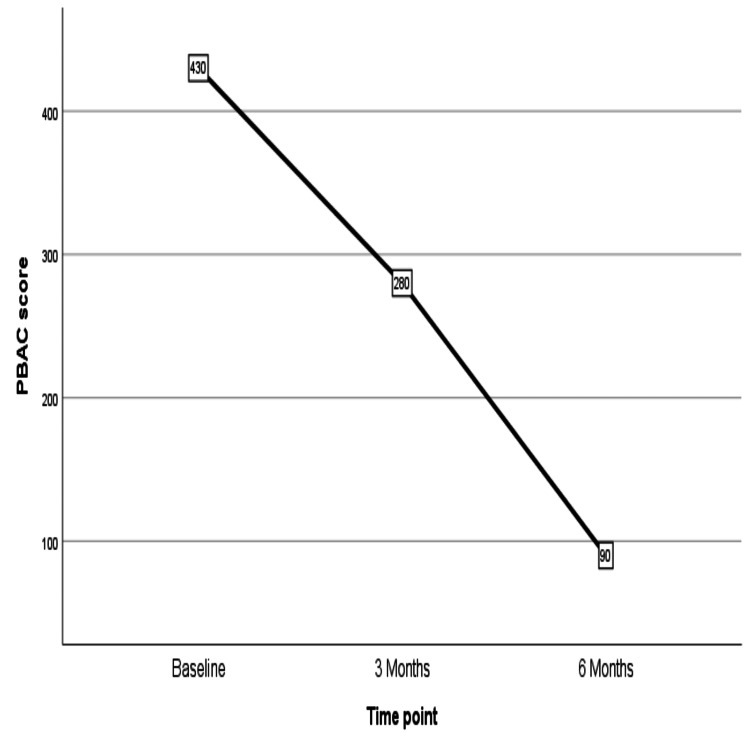
Fall in median PBAC from 430 to 90 over six months

**Figure 5:**
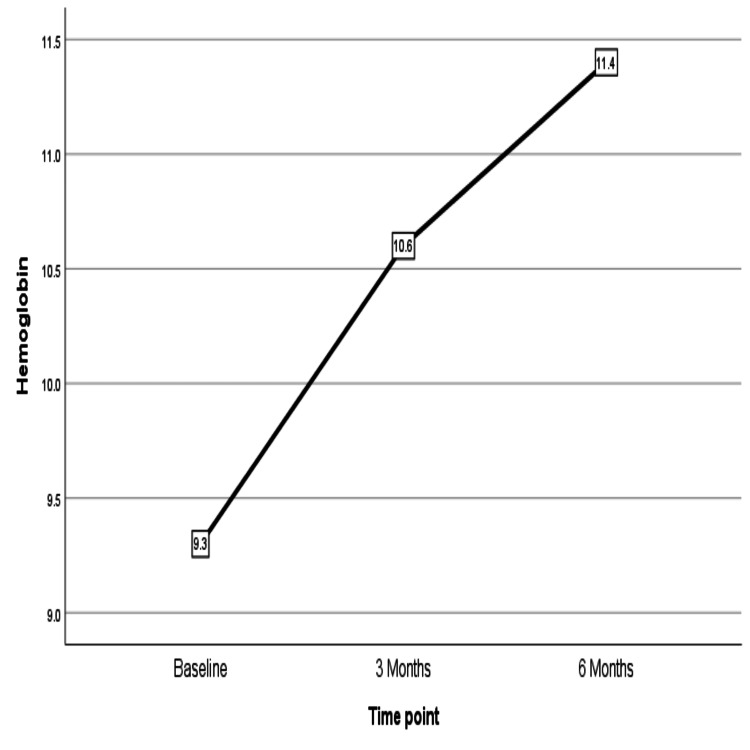
Rise in hemoglobin from 9.3 gm/dL to 11.4 gm/dL over six months from baseline values. Both these changes were statistically very significant (p-value=0.000)

**Figure 6:**
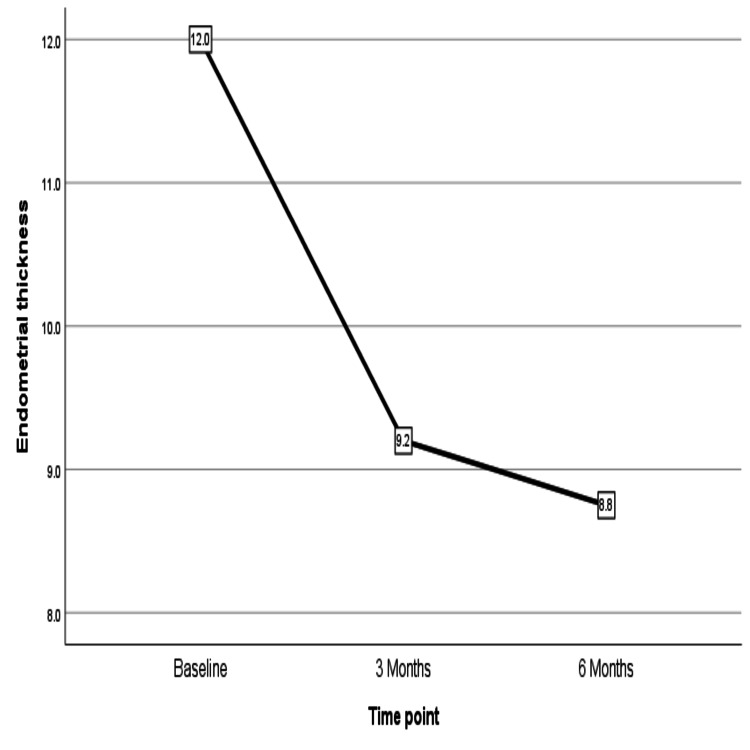
Change in the median value of the endometrial thickness from 12.0 mm to 8.8 mm over six months

The demographic details of the patients are included in Table 1. Two women had diabetes and hypertension, while one woman was diabetic. Five women had a BMI above 25 kg/m^2^, and one was morbidly obese. The chronic menstrual blood loss led to anemia in most of the women, with five women having severe anemia who had hemoglobin less than 7 gm/dL. Within three months, PBAC scores reduced significantly, which continued to reduce over six months of use from the time of insertion (Figure 4). There was a corresponding increase in hemoglobin as menstrual loss reduced over six-month periods (Figure 5). Figure 6 shows a reduction in endometrial thickness from baseline to the third and sixth months of use.

**Table 1:** Baseline demographic characteristics of participants

CONTINUOUS VARIABLES			
		Median; IQR	Range
1	Age (in years)	43;10.5	27-50
2	BMI (in kg/m^2^)	23.4; 1.9	21.6-39.3
3	PBAC score	430; 200	275-825
4	Hemoglobin (gm/dL)	9.3; 1.2	4.3-11.7
5	Serum TSH (IU/L)	2.32; 1.2	0.77-6.07
6	Endometrial thickness (in mm)	12; 15.5	8-24
DISCRETE VARIABLES			
			Number
1	Parity: 2, 3		20, 5
2	Disordered proliferative endometrium, endometrial hyperplasia without atypical, secretory endometrium, endometrial breakdown		15, 4, 4, 2

**Figure 7:**
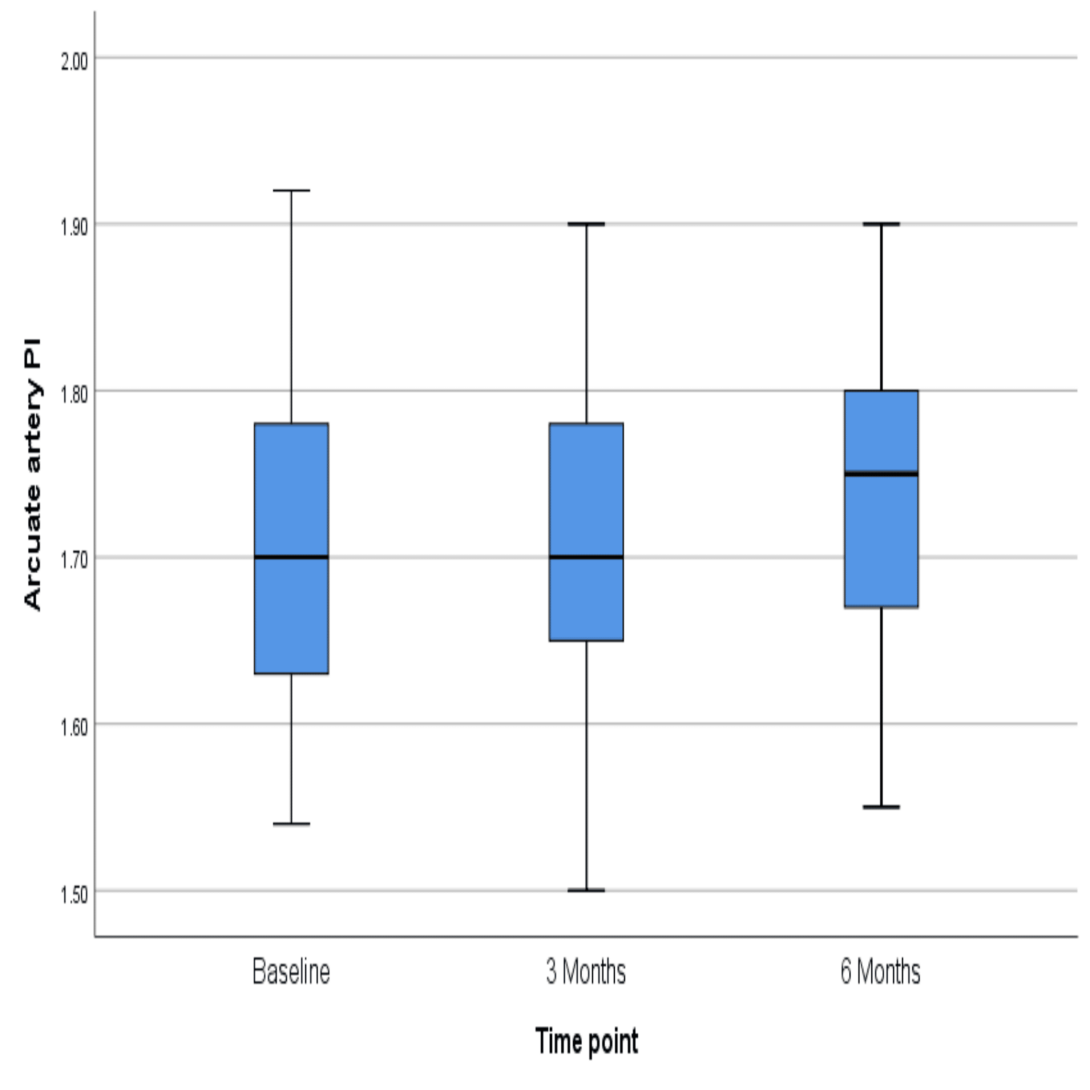
Change in the median value of the pulsatility index in the arcuate artery over six months

**Figure 8:**
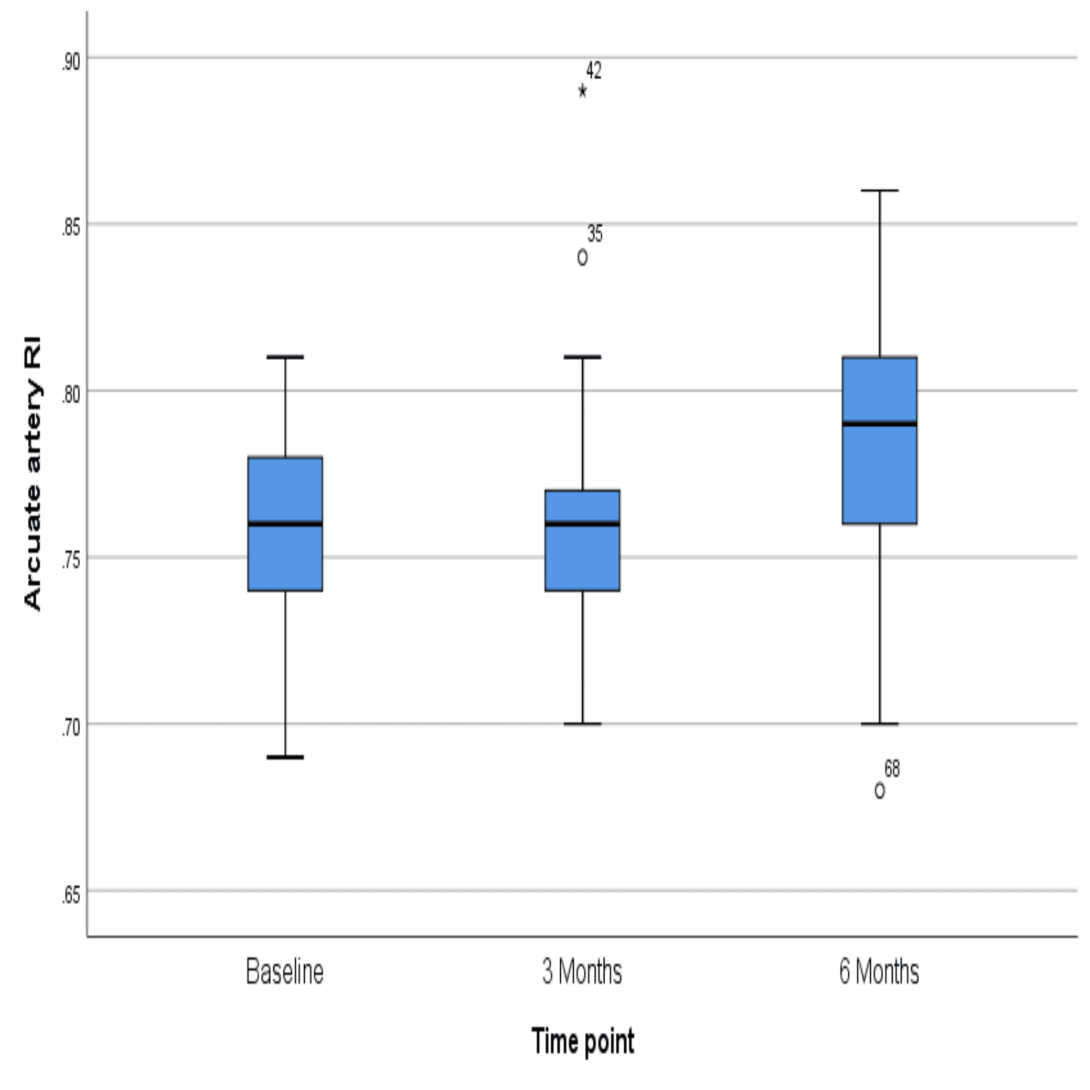
Change in the median value of the resistive index in the arcuate artery

**Figure 9:**
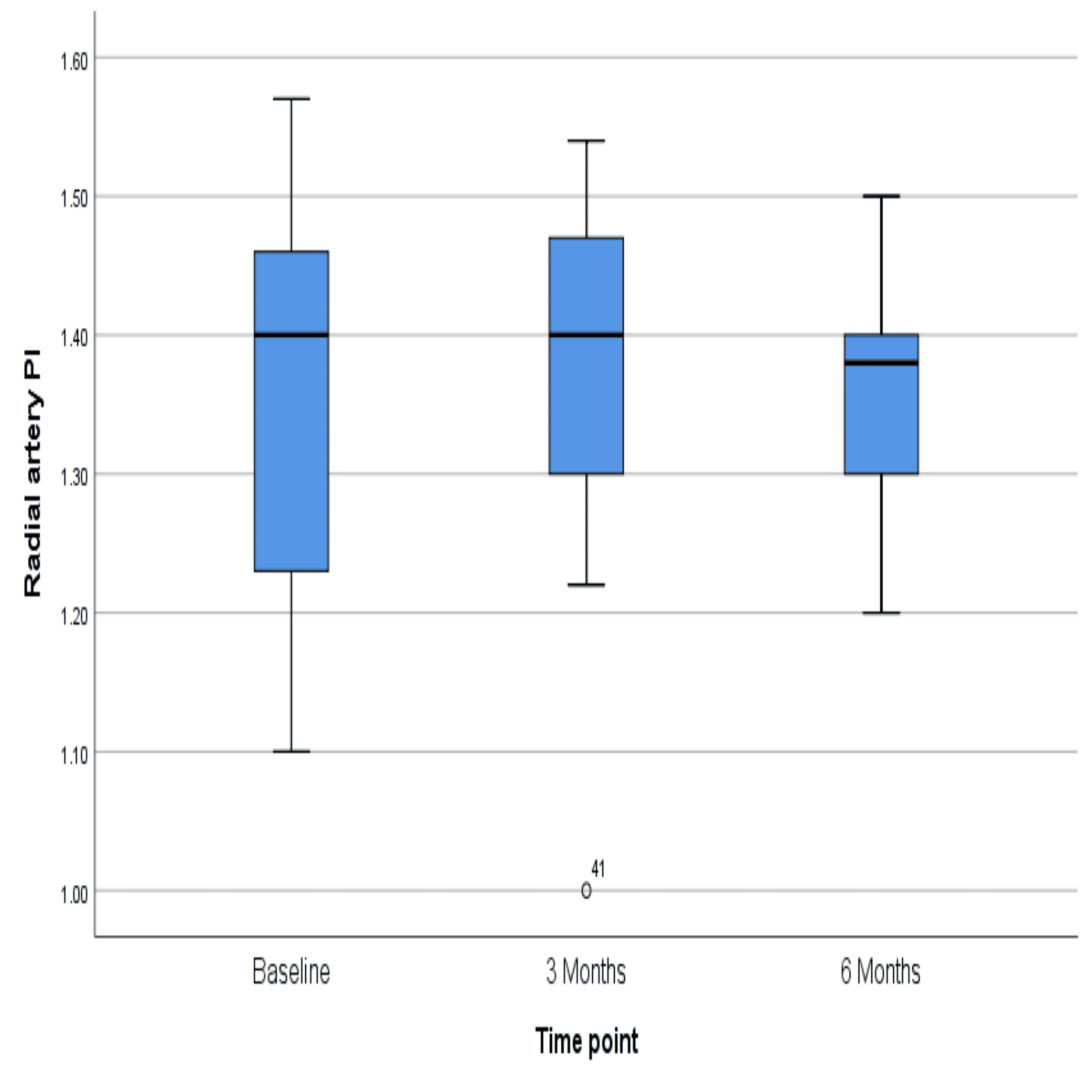
Change in the median value of the pulsatility index in the radial artery over six months

**Figure 10:**
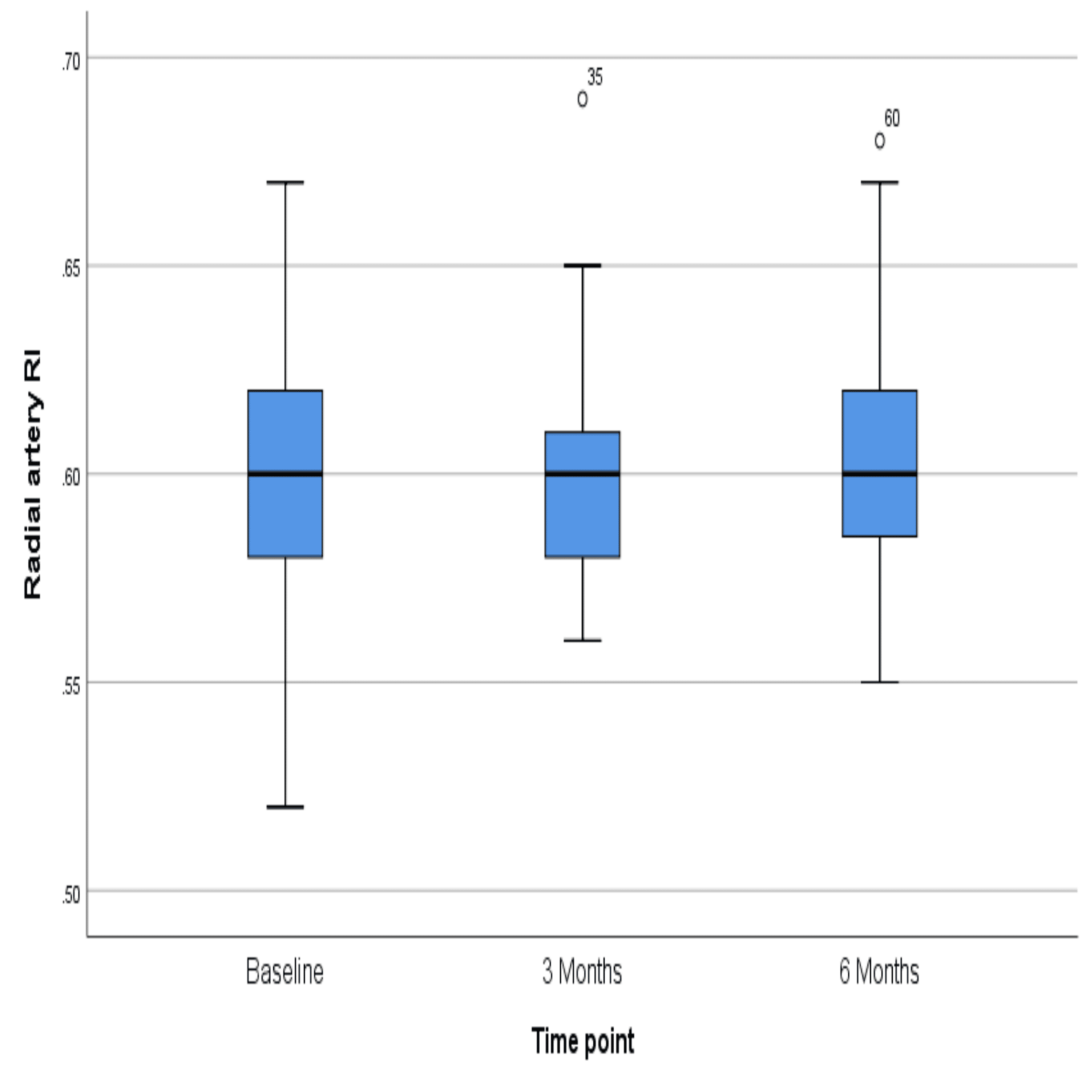
Change in the median value of the resistive index in the radial artery

The PI of the arcuate artery did not change much over six months, the p-value being 0.43 (Figure 7), but the resistive index showed a significant rise over six months (p = 0.03) (Figure 8). However, similar changes were not reflected in the radial artery, for which PI and RI did not change significantly (p = 0.39 and 0.13, respectively) (Figures 9-10). The exact values are shown in Table 2.

**Table 2:** Doppler parameters of the arcuate and radial artery

Arcuate artery		
	Pulsatility index	Resistive index
Baseline	1.7	0.76
3 months	1.7	0.76
6 months	1.75	0.79
P value	0.43	0.03
Radial artery		
	Pulsatility index	Resistive index
Baseline	1.4	0.6
3 months	1.4	0.6
6 months	1.38	0.6
P value	0.39	0.13

## Discussion

Abnormal uterine bleeding is defined as bleeding from the uterine corpus that is abnormal in regularity, volume, frequency, or duration in the absence of pregnancy [6]. It is the most commonly encountered problem in gynecological OPD and can affect 10-30% of women in the reproductive age group and may affect 50% of peri-menopausal women [7-9].

LNG-IUS is an excellent alternative to surgery providing long-term relief, and the patient has the advantage of not taking oral drugs daily [10]. LNG-IUS significantly reduces menstrual blood loss by reducing endometrial thickness [11]. The reduction in menstrual blood loss helps in improving hemoglobin. Due to a significant decrease in PBAC score and endometrial thickness, most of the patients were quite satisfied with the treatment and continued the use of LNG-IUS, except for three women who did not respond to LNG-IUS treatment. In the women who continued the use of LNG-IUS, there was a significant improvement in hemoglobin values over six months [12]. The PI of the arcuate artery remains unchanged, while the resistive index increases over six months; however, similar changes are not observed in the radial artery doppler, which is a branch of the arcuate artery. Thus, this increase in resistive index might be statistically significant but not clinically relevant. Additionally, there was no difference in Doppler findings in patients who did not respond to LNG-IUS treatment.

The endometrium has an opposite response to estrogen as compared to progesterone. Progesterone induces secretory changes in epithelial cells and inhibits their growth. There is also decidualization of the stroma [13]. Since the effect on endometrial thickness is known to be inhibitory, there is a hypothesis that the reduction in vascularity might be responsible for this atrophy. The first study, which evaluated the effect of LNG-IUS on uterine artery Doppler, was done in 1995 by Pakarinen et al. who found no changes in PI three months after the insertion of LNG-IUS, in 10 women for whom LNG-IUS insertion was done for the contraceptive purpose [14].

Zalel et al. compared sub-endometrial flow in women using copper-IUD and LNG-IUS and concluded that LNG-IU causes a significant reduction in sub-endometrial flow, whereas copper-IUD does not [3]. However, there was no significant change in the cervical branch of the uterine artery. However, in a similar study by Jiménez et al., there was no difference in sub-endometrial vascularization between the groups [15].

It was Haliloglu et al. who compared two similar groups of 60 women after one year of insertion [16]. There was no change in Cu-IUD users, whereas RI was significantly higher in LNG-IUS users after one year. Bastianelli et al. found a significantly lower PI and RI in women with LNG-IUS at six months follow-up who had either normal cycles or prolonged bleeding [17].

In all the above previous studies, the endometrial thickness was reduced significantly; however, the effect on the spiral artery is unchanged, and the effect on sub-endometrial vascularisation is inconclusive. In the present study, there was a significant reduction in endometrial thickness and no effect on PI after six months of use. RI of the arcuate artery was increased at six months of use, which was not observed in the radial artery, which is a branch of the arcuate artery. Although the change in RI of the arcuate artery is statistically significant, it does not seem to be clinically significant. Hence, we cannot conclude that LNG has a substantial effect on intra-myometrial vascularity.

## Conclusions

After six months of LNG-IUS, there is a significant reduction in endometrial thickness and menstrual flow. There is no change in the PI of the arcuate and radial arteries after six months of use. The RI showed an increase in the arcuate artery but not the radial artery, which might not be clinically significant.
